# LncRNA Nqo1-AS1 Attenuates Cigarette Smoke-Induced Oxidative Stress by Upregulating its Natural Antisense Transcript Nqo1

**DOI:** 10.3389/fphar.2021.729062

**Published:** 2021-09-08

**Authors:** Haiyun Zhang, Ruijuan Guan, Zili Zhang, Defu Li, Jingyi Xu, Yuxin Gong, Xin Chen, Wenju Lu

**Affiliations:** ^1^ Department of Pulmonary and Critical Care Medicine, Zhujiang Hosptial, Southern Medical University, Guangzhou, China; ^2^ State Key Laboratory of Respiratory Diseases, The First Affiliated Hospital of Guangzhou Medical University, Guangzhou, China

**Keywords:** COPD, lncRNA, Nqo1 antisense transcript 1 (Nqo1-AS1), oxidative stress, cigarette smoke

## Abstract

Evidence of the involvement of long noncoding RNAs (lncRNAs) in the pathogenesis of chronic obstructive pulmonary disease (COPD) is growing but still largely unknown. This study aims to explore the expression, functions and molecular mechanisms of Fantom3_F830212L20, a lncRNA that transcribes in an antisense orientation to Nqo1.We name this lncRNA as Nqo1 antisense transcript 1 (Nqo1-AS1). The distribution, expression level and protein coding potential of Nqo1-AS1 were determined. The effects of Nqo1-AS1 on cigarette smoke (CS)-induced oxidative stress were also evaluated. The results showed that Nqo1-AS1 were mainly located in the cytoplasm of mouse alveolar epithelium and had a very low protein coding potential. Nqo1-AS1 (or its human homologue) was increased with the increase of CS exposure. Nqo1-AS1 overexpression enhanced the mRNA and protein levels of Nqo1 and Serpina1 mRNA expression, and attenuated CS-induced oxidative stress, whereas knockdown of Nqo1-AS1 significantly decreased Nqo1 and Serpina1 mRNA expressions, and aggravated CS-induced oxidative stress. Nqo1-AS1 increased Nqo1 mRNA stability and upregulated Nqo1 expression through antisense pairing with Nqo1 3′UTR. In conclusion, these results suggest that Nqo1-AS1 attenuates CS-induced oxidative stress by increasing Nqo1 mRNA stability and upregulating Nqo1 expression, which might serve as a novel approach for the treatment of COPD.

## Introduction

Chronic obstructive pulmonary disease (COPD) is a lung disease that is usually progressive degenerative and characterized by persistent respiratory symptoms and incompletely reversible expiratory airflow limitation (Duffy and Criner, 2019; Gu et al., 2021), which is a leading cause of death and disability worldwide (Wang et al., 2018; Riley and Sciurba, 2019). Cigarette smoke (CS)-induced oxidative stress is one of the most important pathogenetic mechanisms involved in pulmonary emphysema and COPD (Lu et al., 2018; Barnes, 2020; Guan et al., 2020). NAD(P)H quinone oxidoreductase 1 (Nqo1), one of the most critical quinone reductases, has been well-documented to play crucial roles in antioxidant protection and tumor-killing (Zhang et al., 2018a; Li et al., 2019). It has been well established that Nqo1 is closely related to CS, CS-induced oxidative stress or obstructive bronchitis. It is demonstrated that NQO1 expression is the activation of aryl hydrocarbon receptor (AhR) pathway by propolis, which promotes lung repair in a mouse emphysema model caused by CS exposure (Barroso et al., 2017).Also, studies have proved that NQO1 P187S polymorphisms is determined as risk genotype in children with obstructive bronchitis, whose mother smoke actively during their pregnancies. Previous studies have reported that Nqo1 expression in lung tissue was upregulated by CS exposure (Adair-Kirk et al., 2008; Shahdoust et al., 2013).Recently, a study showed that overexpression of Nqo1 was able to increase scavenging of superoxide in Chinese hamster ovary cells, suggesting that Nqo1 plays a critical role in antioxidant protection (Ross and Siegel, 2017). However, the role of Nqo1 in COPD is still unknown.

Long non-coding RNAs (lncRNAs) are a class of transcripts with length of more than 200 nucleotides but without protein-coding capacity. Through epigenetic modification, control of transcription, RNA processing and translation, lncRNAs have been shown to play crucial roles in various biological processes such as cell growth, metabolism, differentiation and apoptosis (Liao et al., 2018). Some lncRNAs have been shown to be correlated with the occurrence and development of COPD. For example, COPDA1 promotes the proliferation of smooth muscle cells through upregulating the expression of MS4A1 in COPD (Zheng et al., 2019). LINC00987 modulates LPS-induced cell apoptosis, oxidative stress, inflammation and autophagy through sponging let-7b-5p in COPD (Wang et al., 2020, Chen, Chen, Liu, Dong, Ji, Hu, Zhang). MEG3 targets miR-218 thereby regulating cigarette smoke extract (CSE)-inhibited proliferation and CSE-induced apoptosis in COPD (Song et al., 2020). MALAT1 exhibits clinical implications in acute exacerbation risk prediction and management of COPD (Liu et al., 2020). However, the expressions and functions of lncRNAs in COPD progression are largely unknown.

In previous study, we reported that lncRNA Fantom3_F830212L20 and Nqo1 were co-expressed lncRNA and protein-coding gene, and both of two were significantly up-regulated in lung tissues of chronic CS-induced COPD mouse model, 16HBE cells and A549 cells exposed to CSE treatment when compared to their controls (Zhang et al., 2018b). In the present study, we identified the characterization of Fantom3_F830212L20, a lncRNA that transcribed in an antisense orientation to Nqo1 and had a very low protein coding potential, which were mainly located in the cytoplasm of alveolar epithelial cells of mouse lung tissues. We named this lncRNA as Nqo1 antisense transcript 1 (Nqo1-AS1). We further proved that Nqo1-AS1 was upregulated in lung tissues of mice exposed to CS and the mle-12 cells treated with CSE, and its human homologue expression was upregulated in peripheral blood mononuclear cells (PBMCs) of patients with COPD when compared to those of the control group. Nqo1-AS1 was able to inhibit CS-induced oxidative stress as indicated by increased levels of malondialdehyde (MDA), glutathione disulfide (GSSG) and reactive oxygen specicies (ROS). Mechanistically, Nqo1-AS1 upregulated Nqo1 expression through binding Nqo1 3′UTR and increasing Nqo1 mRNA stability thereby attenuating CS-induced oxidative stress. This study provides new insights into the therapy strategy for the treatment of COPD.

## Materials and Methods

### Animal Experiments

C57BL/6J male mice (6–8 week old) were purchased from Guangdong Medical Laboratory Animal Center (Guangzhou, China). Mice were randomly divided into two groups. One group was exposed to CS generated from 9 filter-tipped cigarettes in a 342 L fume chamber (60 cm × 57 cm × 100 cm) each time, twice a day, 6 days per week. Each CS exposure was lasted for over 2 h per time with the interval between two CS exposures more than 4 h. To better demonstrate the effect of CS on the expression levels of Nqo1 and Nqo1-AS1 in lung tissues of mice, mice in the CS group were exposed to CS for 1 week, 1 month and 3 months. Moreover, chronic CS-induced COPD mouse model was constructed as we did before in order to elucidate the distribution of Nqo1-AS1 in lung tissues of mice (Zhang et al., 2018b). The Red Roses cigarettes (manufactured from Guangdong Cigarette Factory) emitting 13 mg tar and 1.3 mg nicotine per cigarette were used in this experiment. Mice in the control group were housed in a smoke-free environment. All experimental procedures were approved by the Animal Care and Use Committee of The First Affiliated Hospital of Guangzhou Medical University.

### Human Samples

A total of seven patients with COPD and seven healthy individuals were recruited between March and May at 2018 in The First Affiliated Hospital of Guangzhou Medical University. Clinical data, including age, smoking information and lung function were collected. Blood samples were obtained with written informed consents from all participants. Whole blood samples were drawn and centrifuged at 1,000×*g* for 15 min. Plasma supernatants were collected and stored at − 80° C until analysis. PBMCs were isolated using lymphocyte separation medium according to the method described previously. This study was approved by the Ethics Committee of The First Affiliated Hospital of Guangzhou Medical University (Ethic Ref No.GZMC 2009-08-1336) and adhered to the Declaration of Helsinki as described previously.

### Protein-Coding Potential Analysis

To verify whether Nqo1-AS1 was able to encode protein, bioinformatics analysis and *in vitro* translation assay were performed as described previously (Liu et al., 2020). Briefly, the RNA sequences of Nqo1-AS1, Nqo1 and Hotair were put into the Coding Potential Calculator http://cpc.cbi.pku.edu.cn/ and Coding-Potential Assessment Tool http://lilab.research.bcm.edu/cpat/index.php. Next, the open reading frame (ORF) sequence of Nqo1-AS1, Nqo1 or Hotair was predicted using the ORF finder database (https://www.ncbi.nlm.nih.gov/orffinder), respectively. Then the predicted ORF sequence of Nqo1-AS1, Nqo1 or Hotair was synthesized and subcloned into the BsrGI and XhoI sites of pcDNA3.1-EGFP vector (Invitrogen). Next, the recombinant plasmid pcDNA3.1-EGFP- Nqo1-AS1 (pc-EGFP- Nqo1-AS1), pcDNA3.1-EGFP-Nqo1 (pc-EGFP-Nqo1) or pcDNA3.1-EGFP- Hotair (pc-EGFP- Hotair) was transfected into the mle-12 cells using Lipofectamine 3000 (Thermo) according to the manufacturer’s instructions, respectively. After transfection for 72 h, the nuclei were stained with DAPI (Beyotime), and the immunofluorescence of cells was observed using a fluorescence microscope. Pc-EGFP-Nqo1 was used as a positive control. Pc-EGFP- Hotair was used as a negative control.

### RNA ISH

Nqo1-AS1 expression was checked by *in situ* hybridization in lung tissues of mouse. The RNA ISH probe mixture of Nqo1-AS1, Gapdh or U6 RNA was synthesized and labeled with digoxigenin from Biosense Bioscience Co. Ltd. (Guangzhou, China). The probe sequences for RNA ISH were as follows: Nqo1-AS1 antisense probe: 5′- TAT​TTA​GGT​GTG​TAT​GCA​TAC​GTG​AGC​CAT​GGC​GCG​CCC​TGT​GGA-3′; Nqo1-AS1 sense probe: 5′- TCC​ACA​GGG​CGC​GCC​ATG​GCT​CAC​GTA​TGC​ATA​CAC​ACC​TAA​ATA -3′; Gapdh: 5′-TAA​GCA​GTT​GGT​GGT​GCA​GGA​TGC​ATT​GCT​GAC​AAT​CTT​GAG​TGA​GTT​GTC​ATA​TTT​CTC GTGGTTCACACCCATCA -3′. The Gapdh or U6 RNA probe was used as a positive control. The Nqo1-AS1 sense probe was used as a negative control. RNA ISH was performed as previously described (Mehta-Mujoo et al., 2019). Briefly, the mouse lung tissues were first fixed and embedded with paraffin. Embedded specimens were sectioned at 4 μm thickness. Then sample sections were incubated in graded alcohols, 3% hydrogen peroxide for 30 min and pre-hybridization solution for 2 h. After that, digoxigenin-labeled probes were added in the hybridization solution and incubated with the sections at 37°C overnight in the dark. Next, the sections were incubated with anti-DIG and horseradish peroxidase and observed. The staining scores were assessed based on both immunostaining intensity and the proportion of positive staining cells. The immunostaining intensity was scored 0–3 as follows: 0 (negative staining), 1 (weak staining), 2 (moderate staining) and 3 (dark staining).The proportion of positive staining cells was evaluated as follows: 0 (no positive cells), 1 (< 10%), 2 (10–50%) and 3 (> 50%). Expression of Nqo1-AS1 was evaluated by the final score that was multiplication of the immunostaining intensity and the proportion of positive staining cells. The final scores were divided into two levels: low expression (≤4) and high expression (> 4).

### Nuclear and Cytoplasmic RNA Fractionation Analysis

Nuclear and cytoplasmic RNA isolation in the mle-12 cells was performed using the Cytoplasmic and Nuclear RNA Purification Kit (Norgen, Belmont, CA, United States) according to the manufacturer’s instruction.

### Cell Line and Cell Culture

The mouse alveolar epithelium mle-12 cells were obtained from Shanghai fuxiang biotechnology co., LTD., (Shanghai, China).The mle-12 cells were cultured in DMEM/F12 supplemented with 2% fetal bovine serum (Gibco,United States), 100 IU/mL peniciliin and 100 μg/ml streptomycin, and maintained in a humidified atmosphere of 5% CO_2_ at 37°C.

### Preparation of Cigarette Smoke Extract

CSE was prepared according to the method described previously (Zhang et al., 2018b). Briefly, two cigarettes (Red Roses, China Tobacco Guangdong Zhongyan Industry CO. Ltd., tar, 13 mg; nicotine, 1.3 mg) without filter were burned and then the smoke was collected and finally bubbled through 10 ml serum-free DMEM medium with the use of a vacuum-pump. The resulting solution was filtered through a 0.22 μm filter to remove particles and bacteria and the pH was adjusted to 7.4. The obtained solution was represented 100% CSE and applied to mle-12 cells within 30 min of preparation.

### Plasmid Construction and Transfection

The full-length (FL) Nqo1-AS1, Nqo1-overlapping region (OL) of Nqo1-AS1, Nqo1-non-overlapping region (NOL) of Nqo1-AS1, FL-Nqo1 mRNA were PCR amplified using the SuperScript® III First-Strand Synthesis System (Invitrogen) and subcloned into the ApaI and NotI , NheI and NotI, NheI and XbaI, Nhel and NotI sites of pcDNA3.1 vector (Invitrogen), named pc-Nqo1-AS1, pc-Nqo1-AS1- OL, pc-Nqo1-AS1-NOL or pc-Nqo1, respectively. The primers used were as follows: pc- Nqo1-AS1:5′-ATAAGAATGCGGCCGCGTTTCTTTGCTTTAGCC-3′ (forward), 5′-TTG​CGG​GCC​CGA​TAG​TTC​TGC​CAT​AAC​AAC-3’ (reverse); pc- Nqo1-AS1-OL: 5′-CTA​GCT​AGC​GAT​GTG​TGA​TGT​ATT​CAT​TTA​TTT​CG-3′ (forward), 5′-ATA​AGA​ATG​CGG​CCG​CGA​TAG​TTC​TGC​CAT​AAC-3′ (reverse); pc- Nqo1-AS1-NOL: 5′-GTT​TCT​TTG​CTT​TAG​CCT​GGC​T-3′ (forward), 5′-AGA​TGG​TGG​AGC​ATG​CCT​TTA​A-3′ (reverse); pc-Nqo1: 5′- CTA​GCT​AGC​AGG​CTC​AGC​TCT​TAC​TAG​CCT​AG-3′ (forward), 5′-ATA​AGA​ATG​CGG​CCG​CGA​TGT​GTG​ATG​TAT​TC-3′ (reverse). The 3′UTR of Nqo1 was PCR amplified using the SuperScript® III First-Strand Synthesis System (Invitrogen) and subcloned into the Xhol and XbaI sites of Dual-Luciferase reporter plasmid pmirGLO vector (Promega), named pmirGLO-Nqo1 3′UTR.The primers used were as follows: 5′-CCG​CTC​GAG​GGA​TTT​TTT​TCC​TAA​CAT​ATA​GTT​AGA​C-3′ (forward), 5′-GCT​CTA​GAG​ATG​TGT​GAT​GTA​TTC​ATT​TAT​TTC​G-3′ (reverse). The pcDNA3.1 empty vector was used as a control. A total of 5  ×  10^5^ mle-12 cells were seeded in 6-well plates, and cultured for 18–24 h, and 80–90% cells were used for transfection. Then transfection was performed on cells using Lipofectamine 3000 (Invitrogen) according to the manufacturer’s instructions. After transfection for 24 h, cells were treated with 0 and 0.5% CSE for 24 h and then harvested for analysis.

### Small Interfering RNA Transfection

siRNA specially targeting Nqo1-AS1 (Nqo1-AS1 siRNA) and scrambled negative control siRNA (siRNA CTL) were synthesized by GenePharma (Shanghai, China).The Nqo1-AS1 siRNA sequence was 5′-GCA​UGU​UGC​UGU​GUG​CCU​ATT-3′ and the siRNA CTL sequence was 5′-UUC​UCC​GAA​CGU​GUC​ACG​UTT-3′. A 20 μM siRNA solution was transfected into the mle-12 cells using HiPerFect Transfection (Qiagen) according to the manufacturer’s instructions. After 24 h, more than 95% of the mle-12 cells were still viable. Cells were then treated with 0 and 0.5% CSE for 24 h prior to being collected, and analyzed.

### ROS Assay

Intracellular ROS was measured using 2, 7-dichlorodihydrofluorescein diacetate (DCFH-DA, Beyotime, Shanghai, China) according to the manufacturer’s recommendation. Briefly, the mle-12 cells were seeded into 96-well plates and incubated with 10 mM DCFH-DA for 20 min at 37°C. Cells were washed with 1 × PBS and resuspended in DMEM. Then, DCF fluorescence was detected using fluorescence spectrophotometer (Thermo, MA, United States). The cells treated with Rosup (50 mg/ml) for 30 min were used as positive controls.

### Measurement of MDA

Concentrations of MDA in the mle-12 cells, the mouse lung tissues and serums from patients with COPD and healthy individuals were measured using MDA Assay kit (Beyotime, Shanghai, China) according to the manufacturer’s instructions. Briefly, the mle-12 cells and the homogenate of mouse lung tissues were lysed in lysis buffers for 30 min on ice, respectively. The lysates were then centrifuged at 10,000×*g* for 10 min at 4°C, and the supernatants were collected. The serums from patients with COPD and healthy individuals were collected as well. After being treated with thiobarbituric acid (TBA) working solution, the supernatants and the serums were heated at 100°C for 15 min and then cooled down and centrifuged at 1,000×*g* for 10 min, respectively. The absorbance was measured spectrophotometrically at 532 nm.

### Measurements of Glutathione and GSSG

The levels of GSH and GSSG in the mle-12 cells, the mouse lung tissues and serums from patients with COPD and healthy controls were measured using GSH and GSSG Assay Kit (Beyotime, Shanghai, China) according to the protocols of manufacturer. Briefly, the mle-12 cells, the mouse lung tissues and the serums from patients with COPD and healthy individuals were treated with protein removal reagent M solution at 4°C for 10 min and then centrifuged at 10,000×*g* for 10 min, respectively. The supernatants were collected. Then the GSH test solution and 0.5 mg/ml NADPH solution were added to a 96-well plate containing a standard solution of GSH or the mentioned supernatants. The absorbance was measured spectrophotometrically at 412 nm. Then the content of reduced GSH or GSSG and the ration of reduced GSH/GSSG were calculated.

### Quantitative Real-Time PCR

The total RNA was extracted from mouse lung tissues, cultured cells or PBMCs from patients with COPD and healthy individuals using Trizol reagent (Invitrogen) and reversely transcribed to cDNA using PrimeScript™ RT reagent Kit (TaKaRa, China). QRT-PCR expression analysis was performed on CFX96–C1000 system (Bio-Rad, CA) using SsoFast™ EvaGreen® supermix kit (Bio-Rad). Primers used for qRT-PCR were as follows: mouse Nqo1-AS1 non-overlapping region (Nqo1-AS1-NOL): 5′-TTG​GAA​TGC​TGA​GAC​CCT​GT-3′ (forward), 5′-GGA​GTG​AAA​ACA​CGT​GGC​TT-3′ (reverse); mouse Nqo1-AS1 overlapping region (Nqo1-AS1-OL): 5′-TCG​GGC​TAG​TCC​CAG​TTA​GA-3′ (forward), 5′-AAG​TTA​GTC​CCT​CGG​CCA​TT-3′ (reverse); mouse Nqo1 non-overlapping region (Nqo1-NOL): 5′-GGA​AGC​TGC​AGA​CCT​GGT​GA-3′ (forward), 5′-CCT​TTC​AGA​ATG​GCT​GGC​A-3′ (reverse); mouse Nqo1 overlapping region (Nqo1-OL): 5′-TCG​GGC​TAG​TCC​CAG​TTA​GA (forward), 5′-AAG​TTA​GTC​CCT​CGG​CCA​TT-3′ (reverse); mouse Gapdh: 5′-AGG​TCG​GTG​TGA​ACG​GAT​TTG-3′ (forward), 5′-GGG​GTC​GTT​GAT​GGC​AAC​A-3′ (reverse); Nqo1-AS1 human homologue: 5′-TAT​GGC​AGA​AGG​GAA​TTG​CT (forward), 5′-GCT​TTG​TAA​TTG​AAA​GCA​AGA​AA (reverse); human NQO1: 5′-GAA​GAG​CAC​TGA​TCG​TAC​TGG​C-3′ (forward), 5′-GGA​TAC​TGA​AAG​TTC​GCA​GGG-3′ (reverse); human GAPDH: 5′-ACA​ACT​TTG​GTA​TCG​TGG​AAG​G-3′ (forward), 5′-GCC​ATC​ACG​CCA​CAG​TTT​C-3′ (reverse).A primer sequence for mouse Nqo1-NOL has been previously described (Amara et al., 2012). Primer sequences of mouse Gapdh, human NQO1 and human GAPDH were retrieved from PrimerBank Database (http://pga.mgh.harvard.edu/primerbank/). The relative expression of each gene was normalized to Gapdh expression and calculated using the 2^−△△Ct^ method.

### RNase Protection Assay and the Infection of Mle-12 Cells With α-amanitin

To detect whether Nqo1-AS1 was associated with Nqo1 mRNA, RNase protection assay was performed as previously described (Xia et al., 2021). Briefly, pc-Nqo1-AS1-OL, pc-Nqo1-AS1-NOL or pcDNA3.1 was cotransfected with pc-Nqo1 into mle-12 cells. After transfection for 48 h, the total RNA was extracted from the cells. RNA sample was digested by DNaseI (Invitrogen) and followed by RNAse A + T cocktail (AM2286, Thermo Fisher Scientific) treatment at 37°C for 30 min. Then, the RNA sample was extracted using RNeasy kits (QIAGEN) and was reversely transcribed to cDNA as described above. Nqo1-AS1 overlapping region of Nqo1 (Nqo1-OL), Nqo1-AS1 non-overlapping region of Nqo1 (Nqo1-NOL) and mouse Gapdh mRNA were amplified by PCR and analyzed by agarose gel electrophoresis. Gapdh PCR product was used as a control. Next, to detect whether Nqo1-AS1 increased Nqo1 mRNA stability, mle-12 cells were transfected with pc-Nqo1-AS1-OL, pc-Nqo1-AS1-NOL or pcDNA3.1 for 24 h, then further exposed to 10 μg/ml α-amanitin (MedChemExpress) for 0 h, 6 h, 12 h, 18 and 24 h. Finally, the cells were harvested and RNA was extracted and analyzed by qRT-PCR.

### Luciferase Reporter Assay

To detect whether Nqo1-AS1 increased the stability of Nqo1 mRNA by binding to its 3′UTR, luciferase reporter assay was performed. Briefly, pmirGLO-Nqo1-3′UTR was cotransfected with Nqo1-AS1 siRNA or siRNA CTL, pc- Nqo1-AS1-OL, pc- Nqo1-AS1-NOL or pcDNA3.1 into mle-12 cells. After transfection for 48 h, cells were harvested and were lysed with lysis buffer. Firefly and Renilla luciferase activities were measured using dual-luciferase reporter assay kit (Promega) according to the manufacturer’s instructions.

### Western Blot

The mle-12 cells and the homogenate of mouse lung tissues were lysed in RIPA lysis buffer with PMSF for 30 min on ice. Total protein concentration was measured using BCA protein assay (Beyotime Biotechnology, China). Protein samples were separated by SDS-PAGE and then transferred to PVDF membranes. The PVDF membranes were blocked with 5% skim milk and then incubated with the primary antibodies NQO1 (1:20,000, Abcam Biotechnology, Cambridge, MA, United States) and β-actin (1:5,000, Abcam Biotechnology, Cambridge, MA, United States) overnight at 4°C. After being washed with TBST, the membranes were incubated with the secondary antibody at room temperature for 2 h. Protein bands were detected with ECL reagents (CoWin Biosciences, China) and then visualized using Tanon 5200 chemiluminescence imaging system (Tanon, Shanghai, China).Scanned images were quantified with Image-Pro 6 software.

### Statistical Analysis

Data were presented as mean ± standard error of the mean (SEM). Normality of the variables was evaluated using a Kolmogorov–Smirnov test. Analysis of parametric variables were performed using the Student t test or one-way ANOVA followed by Bonferroni correction for multiple comparisons, while the analysis of non-parametric variables were performed using the chi-square test. All statistical analyses were performed using SPSS version 13.0 software. The correlation between Nqo1-AS1 human homologues expression and the smoking amount of patients with COPD or healthy individuals was evaluated by Pearson’s correlation. A *p*-value less than 0.05 was regarded as statistically significant.

## Results

### Characterization of the Nqo1 Antisense Transcript 1 About Gene Location, Distribution and Protein Coding Potential

**FIGURE 1:**
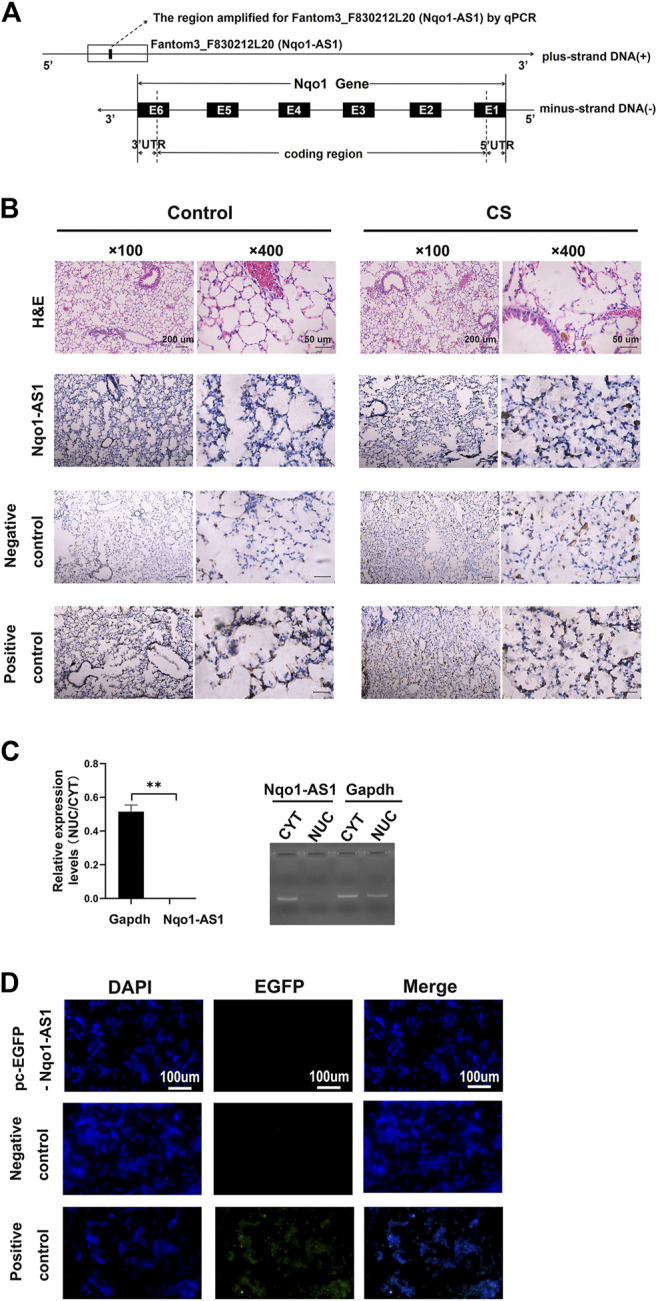
Characterization of lncRNA Fantom3_F830212L20 (Nqo1-AS1) about gene location, distribution and protein coding potential. **(A)** Fantom3_F830212L20 (Nqo1-AS1) located next to the Nqo1 gene on mouse chromosome 8. Nqo1-AS1 was encoded by the (+) DNA strand, while Nqo1was encoded by the (-) DNA strand. Nqo1-AS1 and Nqo1 formed a “tail to tail” pairing pattern with 460 bp full complementarity. **(B)** Representative images of H&E and RNA ISH staining for Nqo1-AS1 in lung tissues of chronic CS-induced COPD mouse model and those of control animals. The expression of Nqo1-AS1 was stronger in lung tissues of chronic CS-induced COPD mouse model than those of control animals. The Nqo1-AS1 sense probe was used as a negative control. The Gapdh or U6 RNA probe was used as a positive control. (Claybank, positive staining). **(C)** Nqo1-AS1 expression in purified nuclear or cytoplasmic RNAs was detected using qPCR. Nqo1-AS1 was enriched in cytoplasm of mle-12 cells. GAPDH served as a control. **(D)** Nqo1-AS1 was verified to have a very low protein coding potential. The recombinant plasmid pcDNA3.1-EGFP- Nqo1-AS1 (pc-EGFP- Nqo1-AS1), pcDNA3.1-EGFP- Hotair (pc-EGFP- Hotair) or pcDNA3.1-EGFP-Nqo1 (pc-EGFP-Nqo1) was transfected into the mle-12 cells for 72 h. Then the immunofluorescence of cells was observed using a fluorescence microscope. Pc-EGFP-Nqo1 was used as a positive control. Pc-EGFP- Hotair was used as a negative control.*p < 0.05 and **p < 0.01. Data represented the mean ± SEM from three independent experiments.

In previous study, we reported that lncRNA Fantom3_F830212L20 and Nqo1 were co-expressed lncRNA and protein-coding gene (Zhang et al., 2018b). To investigate the association between genome loci of Fantom3_F830212L20 and Nqo1, bioinformatics analysis was performed. The results showed that Fantom3_F830212L20 oriented in antisense direction with respect to Nqo1, which formed a “tail to tail” pairing pattern with 460 bp full complementarity between each other. We named this lncRNA as Nqo1 antisense transcript 1 (Nqo1-AS1) (Figure 1A).RNA ISH revealed that the majority of Nqo1-AS1 expression existed in alveolar epithelial cells of mouse with chronic CS exposure, whereas the positive staining was occasionally observed in mouse without CS exposure (Figure 1B). Moreover, subcellular fractionation assay shown that Nqo1-AS1 mainly located in the cytoplasm of mouse alveolar epithelium (Figure 1C). To verify whether Nqo1-AS1 had a coding potential, bioinformatics analysis and an *in vitro* translation assay were performed as described previously (Zhang et al., 2017). The Coding Potential Caculator computational algorithm predicted that Nqo1-AS1 had a very low protein coding potential, similar to Hotair, a well-known lncRNA, whereas Nqo1 was predicted to code for protein (Table 1).Then the recombinant plasmid pc-EGFP- Nqo1-AS1 with the predicted ORF sequence of Nqo1-AS1 was overexpressed in the mle-12 cells (Table 2). Pc-EGFP-Nqo1 was used as a positive control, and pc-EGFP- Hotair was used as a negative control. Immunofluorescence staining displayed that EGFP was hardly detected in cells transfected with pc-EGFP- Nqo1-AS1 or pc-EGFP- Hotair, whereas it was easily detectable in cells transfected with Pc-EGFP-Nqo1. These results suggest that Nqo1-AS1 mainly locates in the cytoplasm of mouse alveolar epithelium and has a very low protein coding potential.

**TABLE 1:** Prediction of protein coding potential for Nqo1-AS1 (Fantom3_F830212L20).

ID	Peptide length	Fickett score	Isoelectric point (pI)	ORF integrity	Coding probability	label
Nqo1-AS1	87	0.32762	5.12103271484	1	0.171312	noncoding
Hotair	48	0.34113	11.539855957	−1	0.184882	noncoding
Nqo1 (NM_008,706.5)	275	0.43184	8.74053955078	1	0.999935	coding

The RNA sequence of Nqo1-AS1, HOTAIR or Nqo1 was put into the Coding Potential Calculator (CPC) algorithm version 2. CPC2 was available freely at http://cpc2.cbi.pku.edu.cn. Hotair was used as a negative control. Nqo1 was used as a positive control.

**TABLE 2:** Prediction of the open reading frame (ORF) sequence of Nqo1-AS1.

ID	Predicted ORF length (bp)	Predicted ORF nucleotide sequence
Nqo1-AS1	261	ATG​TCA​AGT​TGT​TTT​TCT​TTG​GTA​GAA​GGC​TAC​CGG​TTT​TCA​TTG​TGG​CAC​TTA​GGA​TTA​TTT​TTA​TAT​GTA​CAC​CGC​TTT​ATT​ATT​TAT​TTA​TTT​ATA​TCT​ACT​TAT​TTA​TTT​ATT​TAT​TTA​TTT​ATA​TTT​ACT​TAT​CTA​TGC​ATG​CAG​TGG​CTG​CAG​GAG​CCA​GAA​GAG​GGC​ACT​GGA​TCC​CTC​GGA​ACT​GGG​GTT​AGA​AAA​GAG​GTT​GGC​CTT​TCT​GAA​GGT​TTT​CTG​CAA​GAG​CCA​ACA​AGT​GCA​CTT​GGC​TGC​TGA
Hotair	294	ATG​GAA​GGG​TTT​TAC​AAG​TCT​GCA​GGG​GAG​TCA​GGG​AGT​AAA​GAA​ATC​GTG​CCC​AGA​TTT​AGA​GAC​AAT​GGT​GAA​AGA​TAC​AGA​AGA​CAG​AAG​AGA​TGG​GGG​CCG​CCC​CAG​CTG​GCA​GGG​AGT​GGA​GCC​AGA​GGC​AGA​AAA​GGA​GAG​AAA​AGT​TTC​CTG​CCA​TCT​TCA​TTA​GTT​GAC​TTC​CCA​GTC​CAC​AGC​CAC​AGC​TTC​CCG​GGG​CTG​CAG​AAT​TCA​CTC​TCA​ATA​AAG​AAA​GGA​GGC​TTA​AAA​AAA​AAA​AAA​AAA​GTC​CTG​TGT​TTA​CAA​GAC​CAG​AAA​TGC​CAG​CGC​TAA
Nqo1 (NM_008706.5)	825	ATG​GCG​GCG​AGA​AGA​GCC​CTG​ATT​GTA​CTG​GCC​CAT​TCA​GAG​AAG​ACA​TCA​TTC​AAC​TAC​GCC​ATG​AAG​GAG​GCT​GCT​GTA​GAG​GCT​CTG​AAG​AAG​AGA​GGA​TGG​GAG​GTA​CTC​GAA​TCT​GAC​CTC​TAT​GCT​ATG​AAC​TTC​AAC​CCC​ATC​ATT​TCC​AGA​AAT​GAC​ATC​ACA​GGT​GAG​CTG​AAG​GAC​TCG​AAG​AAC​TTT​CAG​TAT​CCT​TCC​GAG​TCA​TCT​CTA​GCA​TAT​AAG​GAA​GGA​CGC​CTG​AGC​CCA​GAT​ATT​GTG​GCC​GAA​CAC​AAG​AAG​CTG​GAA​GCT​GCA​GAC​CTG​GTG​ATA​TTT​CAG​TTC​CCA​TTG​CAG​TGG​TTT​GGG​GTG​CCA​GCC​ATT​CTG​AAA​GGC​TGG​TTT​GAG​AGA​GTG​CTC​GTA​GCA​GGA​TTT​GCC​TAC​ACA​TAT​GCT​GCC​ATG​TAC​GAC​AAC​GGT​CCT​TTC​CAG​AAT​AAG​AAG​ACC​TTG​CTT​TCT​ATC​ACC​ACT​GGG​GGT​AGC​GGC​TCC​ATG​TAC​TCT​CTT​CAG​GGT​GTC​CAC​GGG​GAC​ATG​AAC​GTC​ATT​CTC​TGG​CCG​ATT​CAG​AGT​GGC​ATC​CTG​CGT​TTC​TGT​GGC​TTC​CAG​GTC​TTA​GAA​CCT​CAA​CTG​GTT​TAC​AGC​ATT​GGC​CAC​ACT​CCA​CCA​GAT​GCC​CGC​ATG​CAG​ATC​CTG​GAA​GGA​TGG​AAG​AAA​CGT​CTG​GAA​ACC​GTC​TGG​GAG​GAG​ACC​CCA​CTC​TAT​TTT​GCT​CCA​AGC​AGC​CTG​TTT​GAC​CTA​AAC​TTT​CAG​GCA​GGA​TTC​TTA​ATG​AAA​AAG​GAA​GTT​CAA​GAG​GAG​CAG​AAG​AAG​AAC​AAG​TTT​GGC​CTC​TCT​GTG​GGC​CAT​CAC​CTG​GGC​AAG​TCC​ATT​CCA​GCT​GAC​AAC​CAG​ATC​AAA​GCT​AGA​AAA​TAA

The predicted ORF sequence of Nqo1-AS1, Hotair or Nqo1 was obtained from the ORF finder database (https://www.ncbi.nlm.nih.gov/orffinder), respectively. Hotair was used as a negative control. Nqo1 was used as a positive control.

The RNA sequence of Nqo1-AS1, HOTAIR or Nqo1 was put into the Coding Potential Calculator (CPC) algorithm version 2. CPC2 was available freely at http://cpc2.cbi.pku.edu.cn. Hotair was used as a negative control. Nqo1 was used as a positive control.

The predicted ORF sequence of Nqo1-AS1, Hotair or Nqo1 was obtained from the ORF finder database (https://www.ncbi.nlm.nih.gov/orffinder), respectively. Hotair was used as a negative control. Nqo1 was used as a positive control.

Nqo1-AS1 human homologue is both positively correlated with smoking amount and Nqo1 mRNA expression in PBMCs of patients with COPD or healthy controls

**FIGURE 2:**
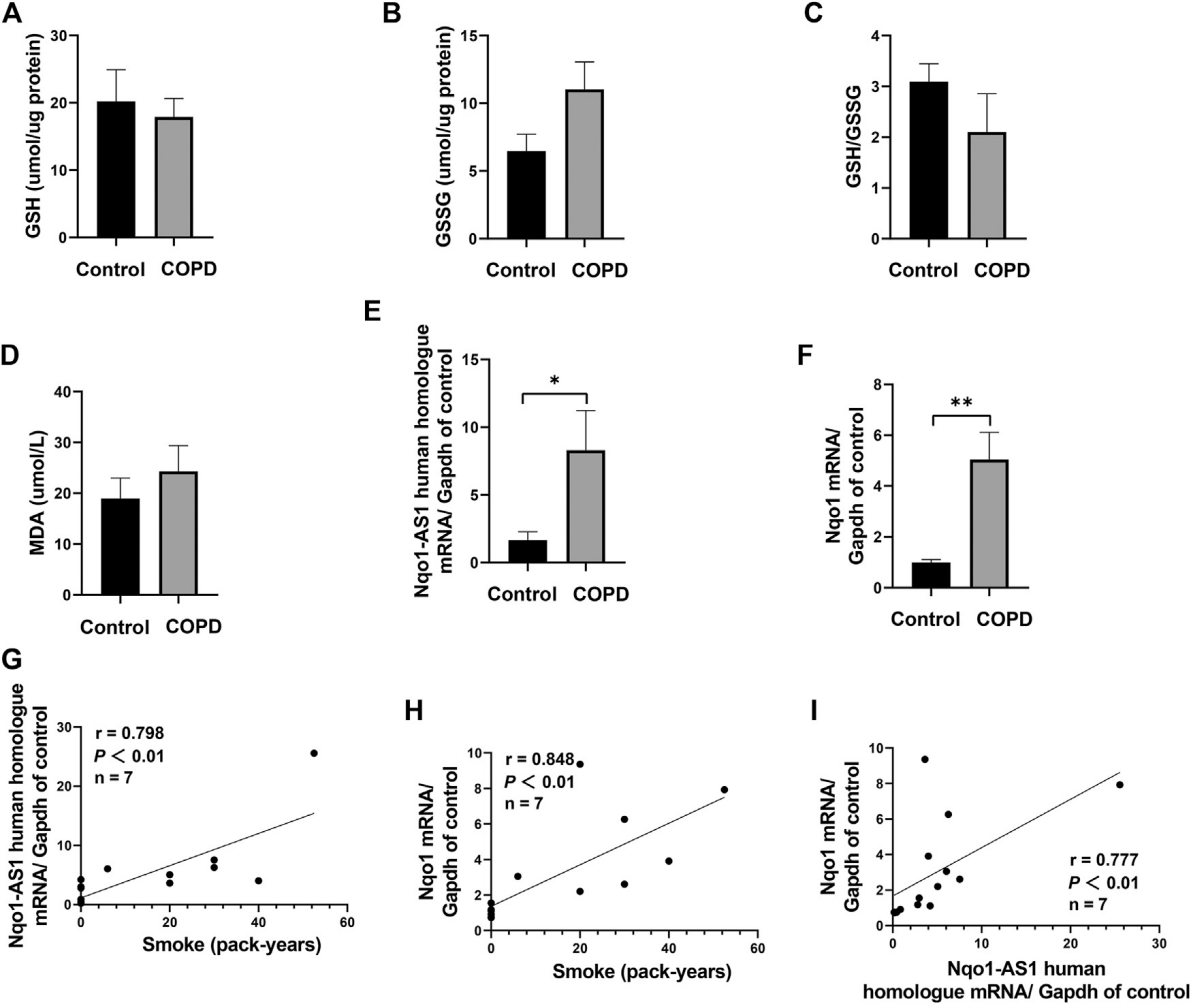
Nqo1-AS1 human homologue is both positively correlated with smoking amount and Nqo1 mRNA expression in patients with COPD. Levels of reduced glutathione (GSH) **(A)**, Glutathione disulfide (GSSG) **(B)**, GSH/GSSG ratio **(C)** and MDA **(D)** were assessed in serums from patients with COPD and healthy controls. Expressions of Nqo1-AS1 human homologue **(E)** and Nqo1 mRNA **(F)** were examined in PBMCs from patients with COPD and healthy controls (*p<0.05; **p <0.01). Both Nqo1-AS1 human homologue **(G)** and Nqo1 mRNA **(H)** expressions were positively correlated with smoking amount of patients with COPD. **(I)** The expression levels of Nqo1-AS1 human homologue and Nqo1 mRNA in PBMCs from patients with COPD and healthy controls were positively correlated with each other (*n* = 7/group; *p* <0.01; r represents spearman correlation coefficient). Nqo1-AS1 is positively correlated with Nqo1 mRNA expression in lung tissue of mice exposed to CS.

To assess whether Nqo1-AS1 human homologue expression was associated with smoking amounts and Nqo1 mRNA expression, the expression levels of Nqo1-AS1 human homologue and Nqo1 mRNA in PBMCs of patients with COPD or healthy controls were examined, and the correlation between these two gene expressions and smoking amounts of patients with COPD or healthy controls were analyzed. A total of seven patients with COPD and seven healthy controls were enrolled in this study. The general characteristics of study participants were summarized in Table 3. As compared to the control group, the GSH concentration and the GSH/GSSG ratio in serum of patients with COPD were lower, whereas concentrations of GSSG and MDA were higher (Figures 2A–D). The expression levels of Nqo1-AS1 human homologue and Nqo1 mRNA in PBMCs from patients with COPD were significantly upregualted than those of the control group (Figures 2E,F). Correlation analysis shown that Nqo1-AS1 human homologue expression was both positively associated with smoking amounts and Nqo1 mRNA expression (Figures 2G–I). These results indicate that Nqo1-AS1 human homologue is both positively associated with Nqo1 mRNA expression and smoking amounts of patients with COPD.

**TABLE 3:** General characteristics of patients with COPD and healthy controls.

	Patients with COPD (*n* = 7)	Healthy controls (*n* = 7)	*p* value
Sex	Male	Male	
Age (year)	64.71 ± 3.06	71.14 ± 4.77	0.28
Smoke (pack-years)	28.36 ± 5.70	0.00	0.00
Height (cm)	162.86 ± 1.10	167.29 ± 1.97	0.07
Weight (kg)	62.86 ± 3.84	70.43 ± 3.27	0.16
BMI (kg/m^2^)	23.66 ± 1.32	25.15 ± 1.05	0.39
FEV_1_ (L)	2.15 ± 0.33	3.31 ± 0.13	0.01
FVC (L)	3.42 ± 0.35	4.08 ± 0.12	0.12
FEV_1_/ FVC (%)	60.28 ± 4.43	82.52 ± 2.28	0.00
FEV_1_%Pred (%)	68.56 ± 8.99	97.26 ± 2.88	0.02

Variables are expressed as mean ± standard error of the mean (SEM). Italicized p values resulted from Student *t* test for parametric variables between the two groups are statistically significant, ie., *p* < 0.05.

BMI, body mass index; FVC, forced vital capacity; FEV1, forced expiratory volume in 1 s; %Pred, percent predicted.

### Nqo1 Antisense Transcript 1 is Positively Correlated With Nqo1 mRNA Expression in Lung Tissue of Mice Exposed to Cigarette Smoke

**FIGURE 3:**
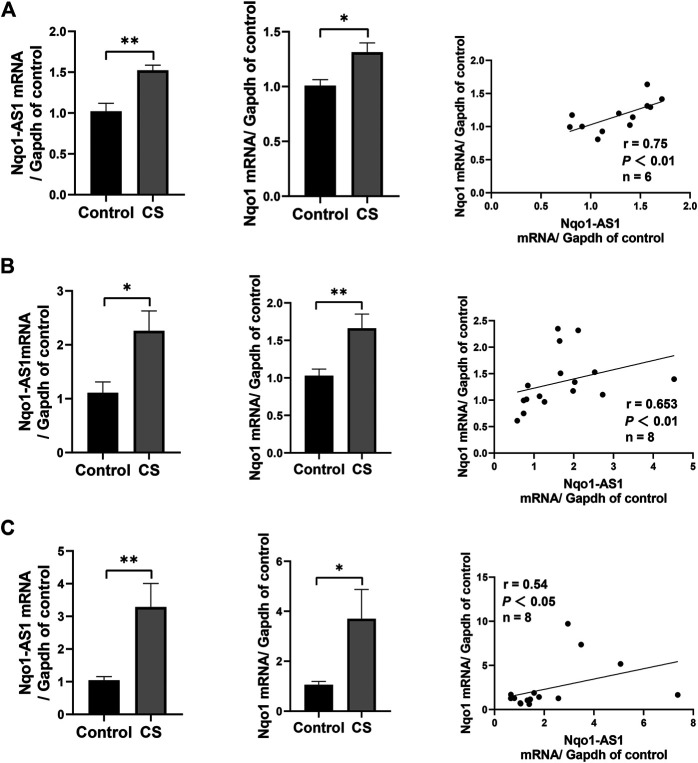
Nqo1-AS1 and Nqo1 mRNA expressions in lung tissues of mice exposed to cigarette smoke (CS) for 1 week **(A)**, 1 month **(B)** and 3 months **(C)**. The expression levels of Nqo1-AS1 and Nqo1 mRNA in lung tissues of mice exposed to CS and those of control animals were measured by qRT-PCR. Expression level of Nqo1-AS1 was positively correlated with Nqo1 mRNA expression in lung tissues of mice with and without CS exposure. *n* = 6/group for **(A)**. *n* = 7/group for **(B)** and **(C)** (*p<0.05; **p <0.01; r represents spearman correlation coefficient).

Given that Nqo1-AS1 human homologue is positively correlated with Nqo1 mRNA expression in PBMCs of patients with COPD, we speculated that Nqo1-AS1 might also be positively associated with Nqo1 mRNA expression in lung tissue of mice exposed to cigarette smoke. QRT-PCR revealed that both Nqo1-AS1 and Nqo1 mRNA expressions were upregulated in lung tissue of mice exposed to CS for 1 week, 1 month and 3 months in comparison with control animals. Correlation analysis shown that Nqo1-AS1 expression was positively associated with Nqo1 mRNA expression in lung tissues of mice (Figures 3A–C). These results suggest that the expression levels of Nqo1-AS1 and Nqo1 mRNA are elevated in lung tissue of mice exposed to CS, and there is a positive correlation between Nqo1-AS1 and Nqo1 mRNA expression.

### Both Nqo1 Antisense Transcript 1 and Nqo1 mRNA Expressions Are Associated With Cigarette Smoke Extract Concentration and Duration in Mle-12 Cells

**FIGURE 4:**
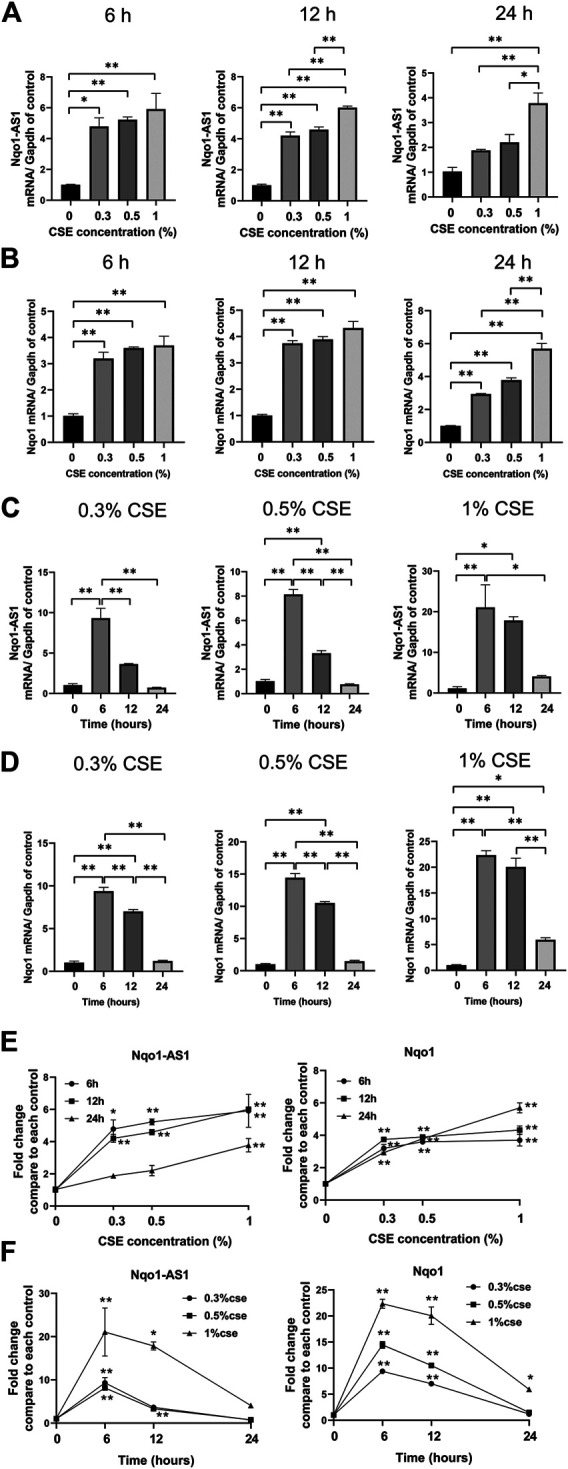
Expression levels of Nqo1-AS1 and Nqo1 mRNA in the mle-12 cells treated with cigarette smoke extract (CSE) at different concentrations for varied time points. Relative mRNA levels of Nqo1-AS1 and Nqo1 were assessed by qRT-PCR. The relative expression of each gene was normalized to Gapdh expression. Relative mRNA levels of Nqo1-AS1 **(A)** and Nqo1 **(B)** were elevated along with the increase of CSE concentration (0–1%) for 6 h, 12 and 24 h. Relative mRNA levels of Nqo1-AS1 **(C)** and Nqo1 **(D)** were elevated predominantly at CSE exposure (0.3–1%) for 6 and 12 h when compared to cells exposed to 0% CSE. **(E)** Line chart shown the trends of Nqo1-AS1 and Nqo1 mRNA expressions in cells exposed to CSE (0–1%) for 6 h, 12 and 24 h exposures (derived from Figures 3A,B). *p < 0.05 and **p < 0.01 vs the cell group treated with 0% CSE. **(F)** Line chart shown the relative mRNA levels of Nqo1-AS1 and Nqo1 were elevated predominantly at CSE exposure (0.3–1%) for 6 and 12 h when compared to cells exposed to CSE (0.3–1%) for 0 h (derived from Figures 3C,D). *p < 0.05 and **p < 0.01 vs the cell group treated with the same CSE concentration for 0 h. Data represented the mean ± SEM from three independent experiments. Statistical significance were indicated (*p<0.05;* *p <0.01) One-way ANOVA and Bonferroni correction for multiple comparisons.

Given that CS exposure was closely associated with the expression levels of Nqo1-AS1 (or its human homologue) and Nqo1 mRNA in lung tissues of mouse or PBMCs from patients with COPD, we speculated that CSE might have effects on the expression levels of Nqo1-AS1 and Nqo1 *in vitro*. The murine alveolar mle-12 cells were treated with CSE at different concentrations for varied time points, and the expression levels of Nqo1-AS1 and Nqo1 mRNA were detected. Compared to the control group, cells treated with 0% CSE, Nqo1-AS1 and Nqo1 mRNA expressions in cells exposed to CSE (0.3–1%) for 6 h, 12 and 24 h were significantly upregulated. Furthermore, Nqo1-AS1 and Nqo1 mRNA expressions were increased after being exposed to CSE in a concentration dependent manner (Figures 4A,B).Additionally, Nqo1-AS1 and Nqo1 mRNA expressions were significantly increased after being exposed to CSE for 6 h, yet both these two gene expressions were gradually decreased in response to the increasing CSE duration (Figures 4C,D). Line chart depicted the trends of Nqo1-AS1 and Nqo1 mRNA expressions in cells exposed to CSE, which elevated with the increase of CSE concentration whereas decreased with the increase of the CSE exposure duration (Figures 4E,F). These results indicate that Nqo1-AS1 and Nqo1 mRNA expressions are closely correlated with CSE concentration, and both of them were elevated mainly in the early stage of exposure of cells to CSE.

### Nqo1 Protein Expression Correlates With Cigarette Smoke Extract Concentration and Duration in Mle-12 Cells

**FIGURE 5:**
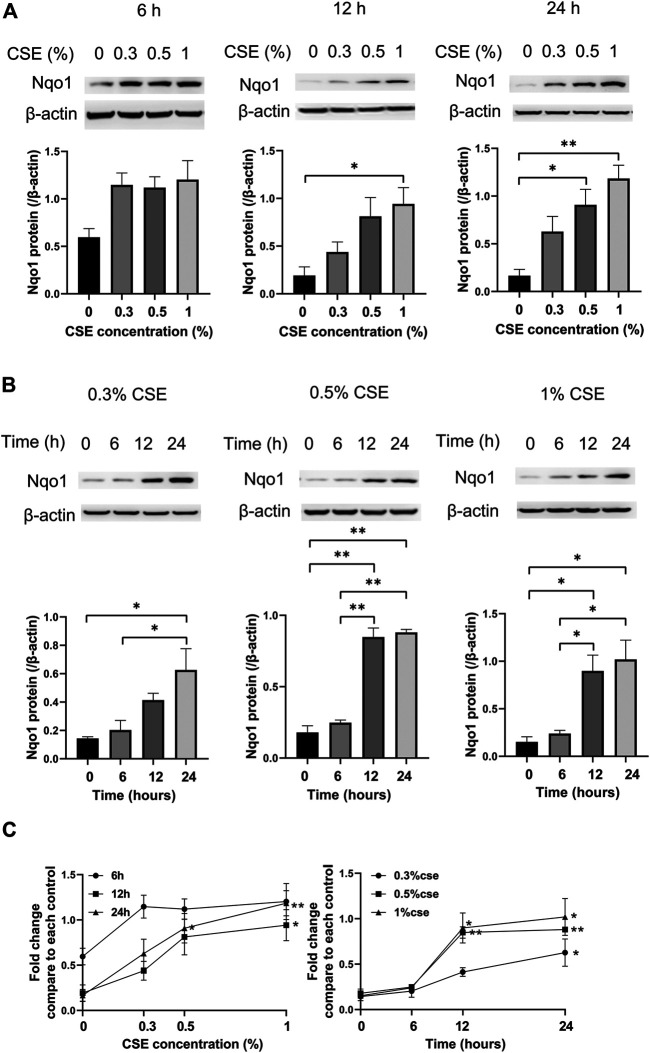
Western blot analysis of Nqo1 expression in the mle-12 cells treated with CSE at different concentrations for varied time points. **(A)** The expression level of Nqo1 protein was elevated along with the increase of CSE concentration (0–1%) for 6 h, 12 and 24 h. **(B)** The expression level of Nqo1 protein was elevated along with the increase of CSE exposure duration. The mel-12 cells were treated with 0.3, 0.5 and 1% CSE respectively. **(C)** Line chart depicted the Nqo1 protein level was increased with the increase of CSE concentration and exposure duration. *p < 0.05 and **p < 0.01. Data represented the mean ± SEM from three independent experiments. One-way ANOVA and Bonferroni correction for multiple comparisons.

Compared to the control group, cells treated with 0% CSE, Nqo1 protein level in mle-12 cells exposed to CSE (0.3–1%) for 6 h, 12 and 24 h were significantly enhanced (Figure 5A). Similarly, compared to cells treated with CSE (0.3–1%) for 0 h, Nqo1 protein level in cells was significantly increased after being exposed to CSE (0.3–1%) for 24 h (Figure 5B). Line chart displayed the trends of Nqo1 protein level in cells exposed to CSE, which was enhanced with the increase of CSE concentration and exposure duration (Figure 5C). These results indicate that CSE enhances Nqo1 protein level of mle-12 in a dose-and time-dependent manner.

### Nqo1-Antisense Transcript 1 Attenuates Cigarette Smoke Extract-Induced Oxidative Stress *in vitro*

**FIGURE 6:**
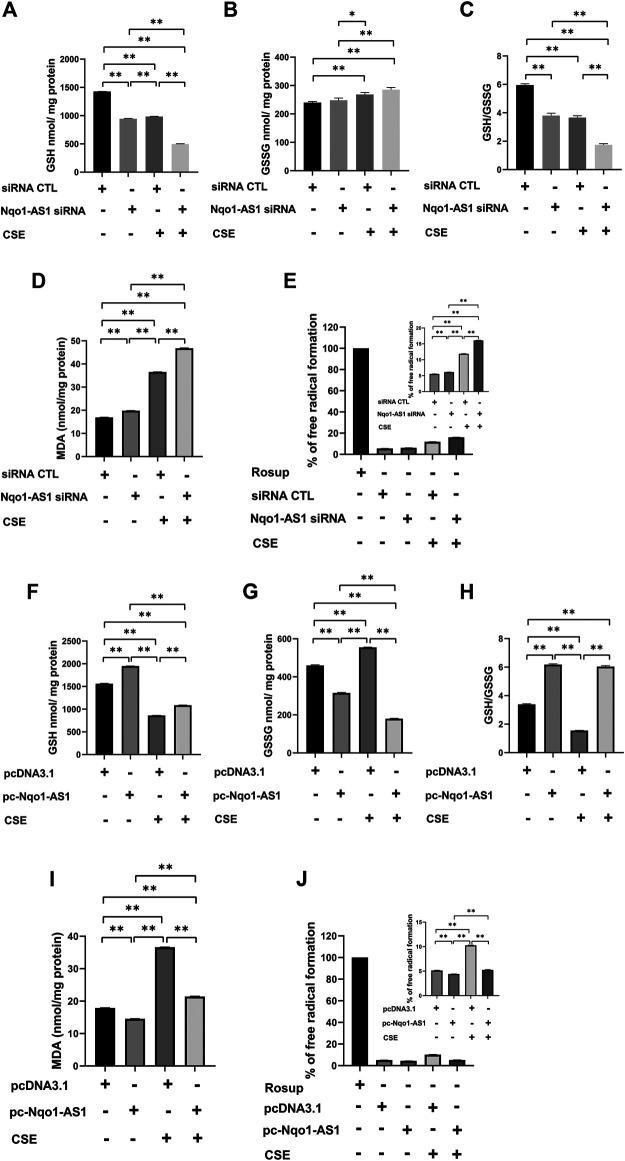
Effect of Nqo1-AS1 on CSE-induced oxidative stress in the mle-12 cells. **(A–E)** Nqo1-AS1 siRNA or siRNA CTL was transfected into the mle-12 cells for 24 h, and followed by 0.5% CSE treatment or medium for 24 h. Then, levels of reduced glutathione (GSH) **(A)**, Glutathione disulfide (GSSG) **(B)**, GSH/GSSG ratio **(C)**, MDA **(D)** and ROS **(E)** were examined in cells with or without CSE treatment. **(F–J)** The pcDNA3.1-Nqo1-AS1 (pc-Nqo1-AS1) or pcDNA3.1 was transfected into the mle-12 cells for 24 h, and followed by 0.5% CSE treatment or medium for 24 h. Oxidative stress indexs such as reduced GSH **(F)**, GSSG **(G)**, GSH/GSSG ratio **(H)**, MDA **(I)** and ROS **(J)** were examined in cells with or without CSE treatment. The cells treated with Rosup (50 mg/ml) for 30 min were used as positive controls. *p < 0.05 and **p < 0.01. Data represented the mean ± SEM from three independent experiments. One-way ANOVA and Bonferroni correction for multiple comparisons.

Accumulating evidences suggest that CS induced oxidative stress plays a critical role in the pathological mechanism of COPD (Kim et al., 2019). To elucidate the effect of Nqo1-AS1 against CSE-induced oxidative stress, mle-12 cells were transfected with Nqo1-AS1 siRNA or siRNA CTL prior to being treated with 0% CSE or 0.5% CSE for 24 h. Then oxidative stress parameters such as GSH, GSSG, MDA and ROS in cells were measured. The cells treated with Rosup (50 mg/ml) were used as positive controls. Compared to cells transfected with siRNA CTL and followed by 0% CSE treatment for 24 h, the GSH content and [GSH/GSSG] ratios in cells transfected with siRNA CTL and followed by 0.5% CSE treatment were significantly reduced, whereas the levels of GSSG, MDA and ROS were significantly enhanced. Knockdown of Nqo1-AS1 worsen the decrease of GSH content and [GSH/GSSG] ratios in cells due to CSE exposure, whereas increasing the levels of GSSG, MDA and ROS (Figures 6A–E). On the contrary, the overexpression of Nqo1-AS1 was able to rescue the decrease of GSH content and [GSH/GSSG] ratios due to CSE, and to alleviate the CSE-increased MDA and ROS in cells (Figures 6F–J). These results lend strong support to our hypothesis that Nqo1-AS1 has a protective effect on CSE-induced oxidative damage to mle-12 cells *in vitro*.

**FIGURE 7:**
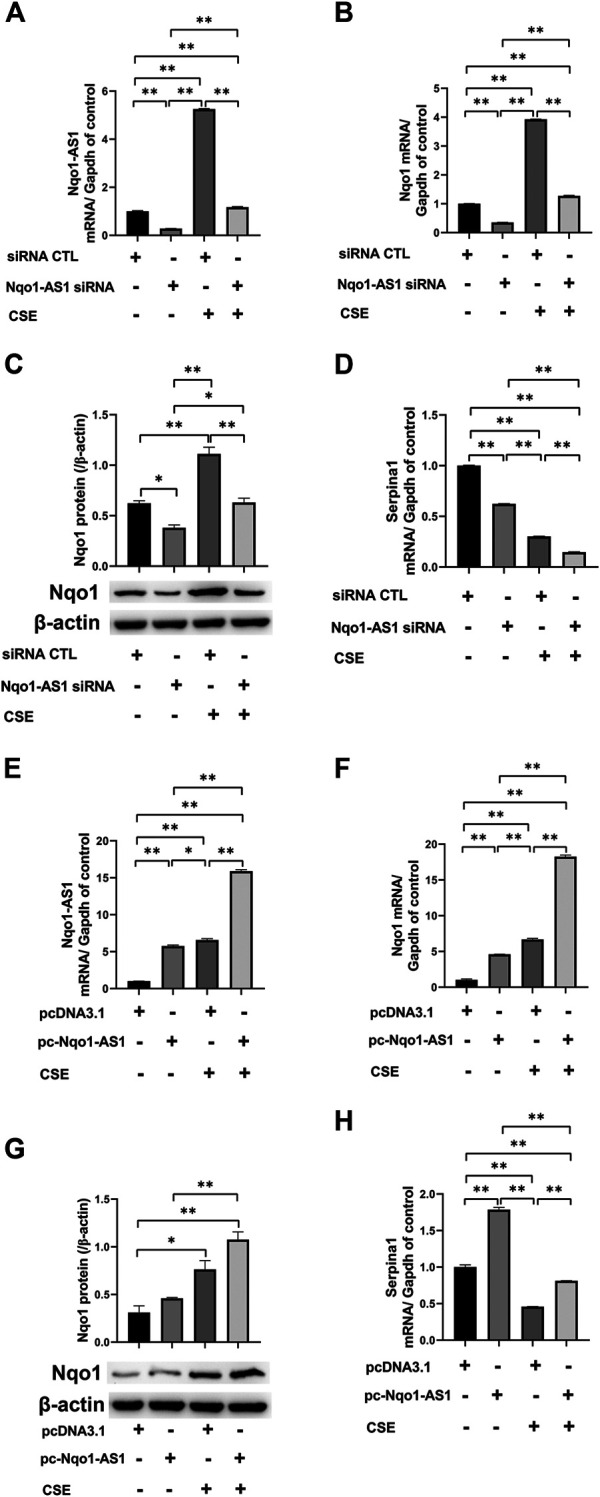
Nqo1-AS1 regulates CSE-induced Nqo1 and Serpina1 expressions in mle-12 cells. **(A)** Nqo1-AS1 siRNA or siRNA control (siRNA CTL) was transfected into the mle-12 cells for 24 h, and followed by 0.5% CSE treatment or medium for 24 h. Next, the interference efficiency of Nqo1-AS1 siRNA was assessed by qRT-PCR. **(B, C)** Nqo1 mRNA **(B)** and protein **(C)** expression levels in cells with or without CSE treatment were examined after knockdown of Nqo1-AS1. The mRNA or protein level of Nqo1 was detected by qRT-PCR or western blotting, respectively. **(D)** Serpina1 mRNA level in cells with or without CSE treatment was measured after knockdown of Nqo1-AS1. **(E)** The pc-Nqo1-AS1 or pcDNA3.1 (control) was transfected into the mle-12 cells for 24 h, and followed by 0.5% CSE treatment or medium for 24 h. Next, the transfection efficiency of pc-Nqo1-AS1 was examined by qRT-PCR. **(F, G)** Nqo1 mRNA **(F)** and protein **(G)** expression levels in cells with or without CSE treatment were examined after overexpression of Nqo1-AS1.The mRNA or protein level of Nqo1 was detected by qRT-PCR or western blotting, respectively. **(H)** Serpina1 mRNA level in cells with or without CSE treatment was measured after overexpression of Nqo1-AS1.*p < 0.05 and **p < 0.01. Data represented the mean ± SEM from three independent experiments. One-way ANOVA and Bonferroni correction for multiple comparisons.

It has been demonstrated that Nqo1 functions as a crucial antioxidant enzyme and is able to bind to Serpina1 mRNA thereby having effect on COPD progression (Di Francesco et al., 2016). We then determined whether Nqo1-AS1 regulated the expressions of Nqo1 and Serpina1 expression. The Nqo1-AS1 siRNA was transfecting into mle-12 cells and the interference efficiency was detected (Figure 7A). QRT-PCR and western blotting shown that knockdown of Nqo1-AS1 expression significantly decreased Nqo1 at mRNA and protein levels. Moreover, down regulation of Nqo1-AS1 inhibited the CSE-induced upregulation of Nqo1 (Figures 7B,C). Interestingly, we also observed that silencing Nqo1-AS1 down-regulated the Serpina1 mRNA expression, and even aggravated the decrease of Serpina1 mRNA expression due to CSE (Figure 7D). To better evaluate the effect of Nqo1-AS1 on the CSE-induced Nqo1 and Serpina1 expressions, we further upregulated the Nqo1-AS1 expression through transfecting pc-Nqo1-AS1 plasmids into the mle-12 cells. The transfection efficiency of pc-Nqo1-AS1 was detected (Figure 7E). As expected, the overexpression of Nqo1-AS1 increased Nqo1 at mRNA and protein levels in cells with or without CSE treatment (Figures 7F,G). In addition, overexpressing Nqo1-AS1 not only enhanced Serpina1 mRNA expression but also rescued CSE-induced downregulation of Serpina1 mRNA (Figure 7H). These results suggest that Nqo1-AS1 is able to regulate CSE-induced Nqo1 and Serpina1 expressions.

### Nqo1-Antisense Transcript1 Increased Nqo1 mRNA Stability and Expression

**FIGURE 8:**
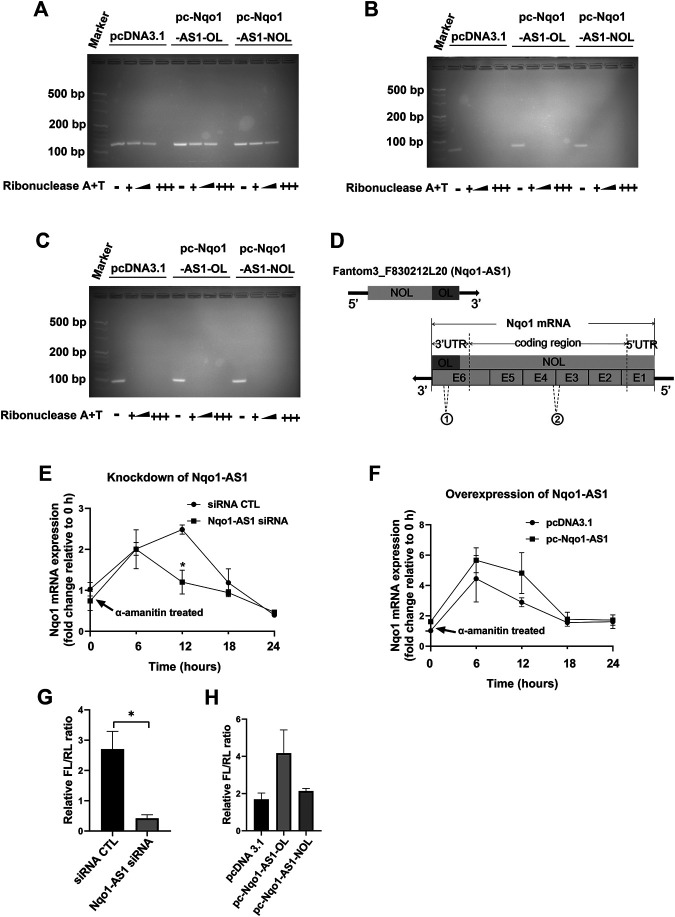
Nqo1-AS1 increases Nqo1 mRNA stability and expression. **(A–C)** RNase protection assay was performed to examine the RNA duplexes formation between Nqo1-AS1 and Nqo1 mRNA. PcDNA3.1-Nqo1-AS1 overlapping region (pc-Nqo1-AS1-OL), PcDNA3.1-Nqo1-AS1 non-overlapping region (pc-Nqo1-AS1-NOL) or pcDNA3.1 (control) vector was cotransfected with pcDNA3.1- Nqo1 (pc-Nqo1) into mle-12 cells. After transfection for 48 h, the total RNA was extracted from the cells. RNA sample was digested with increasing amounts of RNAse A + T cocktail (represented as the black wedge and multiple “+++”) in various samples.Then the remaining double-stranded RNA was reversely transcribed to cDNA and amplified the overlapping part of Nqo1 (Nqo1-OL) mRNA **(A)** and the non-overlapping part of Nqo1 (Nqo1-NOL) mRNA **(B)** by PCR. Gapdh PCR product **(C)** was used as a control. **(D)** Schematic diagram displayed the RNA duplexes formation between the overlapping part of Nqo1-AS1 (Nqo1-AS1-OL) and the overlapping part of Nqo1 (Nqo1-OL) mRNA, which protected both of them from RNase digestion. “E” followed by number represented exon. The sites of primers used in RNase protection assay were indicated as follows:1 Nqo1-OL PCR primer; 2Nqo1-NOL PCR primer. **(E–F)** Line chart shown the stability of Nqo1 mRNA over time relative to time 0 after blocking new RNA synthesis with a-amanitin treatment (10 μg/ml). 18S rRNA was used as an internal control, which was a product of RNA polymerase I and was unchanged after a-amanitin treatment. **(E)** Nqo1-AS1 siRNA or siRNA CTL was transfected into the mle-12 cells for 24 h, and followed by a-amanitin treatment for 0 h, 6 h, 12 h, 18 and 24 h. Then the Nqo1 mRNA expression level was measured by qRT-PCR. **(F)** The pc-Nqo1-AS1 or pcDNA3.1 vector was transfected into the mle-12 cells for 24 h and then treated with a-amanitin for 0 h, 6 h, 12 h, 18 and 24 h. Subsequently, the Nqo1 mRNA expression level was detected by qRT-PCR. **(G)** The luciferase activity of pmirGLO-Nqo1 3′UTR was markedly decreased in the mle-12 cells transfected with Nqo1-AS1 siRNA compared to cells transfected with siRNA CTL. **(H)** The luciferase activity of pmirGLO-Nqo1 3′UTR was increased significantly in the mle-12 cells that overexpressing the Nqo1-OL of Nqo1-AS1, but not the Nqo1-NOL of Nqo1-AS1. *p < 0.05. Data represented the mean ± SEM from three independent experiments.

Since bioinformatics analysis indicated that Nqo1-AS1 was able to form RNA-RNA hybrid with Nqo1 mRNA (Table 4), and Nqo1-AS1 and Nqo1 formed a “tail to tail” pairing pattern with 460 bp full complementarity between each other, we then determined whether Nqo1-AS1 was physically associated with Nqo1 mRNA. RNase protection assay shown that Nqo1-AS1-OL, but not Nqo1-AS1-NOL, protected the overlapping part of Nqo1 (Nqo1-OL) mRNA from RNase digestion by forming the RNA duplexes between Nqo1-AS1 and Nqo1 mRNA, whereas the non-overlapping part of Nqo1 (Nqo1-NOL) mRNA and Gapdh mRNA was totally digested (Figures 8A–C). Gapdh PCR product was used as a control. It was the RNA duplexes formation between the overlapping part of Nqo1-AS1 (Nqo1-AS1-OL) and the overlapping part of Nqo1 (Nqo1-OL) mRNA that protected both of them from RNase digestion (Figure 8D).To further determine whether Nqo1-AS1 regulated Nqo1 mRNA stability, we silenced the Nqo1-AS1 expression by transiently transfecting Nqo1-AS1 siRNA and followed by a-amanitin treatment to block new RNA synthesis in the mle-12 cells. QRT-PCR analysis revealed that knockdown of Nqo1-AS1 decreased Nqo1 mRNA stability (Figure 8E).On the contrary, overexpression of Nqo1-AS1 by transfecting pc- Nqo1-AS1 plasmids into the mle-12 cells increased the stability of Nqo1 mRNA (Figure 8F). As the overlapping region of Nqo1-AS1 antisense paired with Nqo1 3′UTR, we constructed a luciferase reporter plasmid that carried the Nqo1 3′UTR, named pmirGLO-Nqo1-3′UTR.A 594-bp fragment of the Nqo1-3′UTR, which contained the entire overlapping region with Nqo1-AS1, was inserted downstream of the luciferase reporter gene in the pmirGLO-Nqo1-3′UTR vector. We examined the effects of Nqo1-AS1 knockdown or overexpression on pmirGLO-Nqo1-3′UTR activity. The results shown that knockdown of Nqo1-AS1 reduced the luciferase activity of pmirGLO-Nqo1-3′UTR, whereas Overexpressing Nqo1-AS1-OL, but not Nqo1-AS1-NOL, increased the luciferase activity of pmirGLO-Nqo1-3′UTR (Figures 8G,H).Taken together, these results demonstrate that Nqo1-AS1 increases the Nqo1 mRNA stability and promotes the expression of Nqo1 through forming RNA-RNA hybrid with Nqo1 mRNA.

**TABLE 4:** Prediction of the potential interaction between Nqo1-AS1 and Nqo1 mRNA.

Query	Target	dG	ndG	Start Position Query	End Position Query	Start Position target	End Position target
Nqo1-AS1	Nqo1 (NM_008706.5)	−500.43	−250.2150	3,113	3,572	1	460

The RNA sequences of Nqo1-AS1 and Nqo1 were put into the lncRNATargets (http://www.cuilab.cn/lnctar) to analyze the potential interaction between each other.

The RNA sequences of Nqo1-AS1 and Nqo1 were put into the lncRNATargets (http://www.cuilab.cn/lnctar) to analyze the potential interaction between each other.

## Discussion

Increasing evidence suggests that lncRNAs play crucial roles in respiratory diseases, including COPD (Poulet et al., 2020). In a previous study, we reported that lncRNA Fantom3_F830212L20 and Nqo1 were co-expressed lncRNA and protein-coding genes, and both of two were significantly up-regulated in lung tissues of chronic CS-induced COPD mouse model, 16HBE cells and A549 cells exposed to CSE when compared to their controls (Zhang et al., 2018b). In this paper, we identified the characterization of Fantom3_F830212L20 about gene location, distribution and protein coding potential, and assessed whether Fantom3_F830212L20 inhibited CS-induced oxidative stress through regulating Nqo1 expression.

To better understand the characterization of fantom3_F830212L20, we evaluated the genomic locations between fantom3_F830212L20 and Nqo1.Interestingly, fantom3_F830212L20 oriented in antisense direction with respect to Nqo1 and formed a “tail to tail” antisense pairing with Nqo1. So we named fantom3_F830212L20 as Nqo1 antisense transcript Ⅰ (Nqo1-AS1). Recently, quite a few of lncRNAs have been reported to serve as natural antisense transcripts (NATs), which transcribe from the opposite strands of their cognate sense genes and play important roles in various diseases (Latgé et al., 2018). It is the subcellular localization of NATs that are closely related to their different mechanisms of biological functions (Bergalet et al., 2020). Normally, nuclear NATs are mainly involved in transcriptional interference, epigenetic modifications, RNA pocessing and alternative splicing, whereas cytoplasmic NATs are mainly involved in RNA stability and/or mRNA translatability (Xu et al., 2019; Ma et al., 2020; Xu et al., 2020). Therefore we examined the subcellular localization and the protein coding potential of Nqo1-AS1. We observed that Nqo1-AS1 were mainly expressed in cytoplasm of alveolar epithelial cells, and had a very low coding potential. These findings suggest that Nqo1-AS1 serves as a NAT of Nqo1, which has low ability to encode for proteins.

Recently, increasing studies have documented oxidative stress to be a major driving mechanism in the pathogenesis of COPD (Magallón et al., 2019). In this study, we first measured the levels of GSH, GSSG and MDA in the serum of patients with COPD and healthy controls, which were important biomarkers of the oxidative and antioxidant balance system. Notably, we observed that the GSH concentration and the GSH/GSSG ratio were lower in serum of patients with COPD, whereas concentrations of GSSG and MDA were higher, which was consistent with the previous studies about COPD (Leelarungrayub et al., 2018). Moreover, we further found that both Nqo1-AS1 human homologue and Nqo1 mRNA were up-regulated in PBMCs of patients with COPD compared to the healthy controls, and both Nqo1-AS1 human homologue and Nqo1 were not only positively correlated with smoking amount of patients with COPD, but also positively correlated with each other. Since the expression levels of Nqo1-AS1 human homologue and Nqo1 mRNA were intimately associated with the CS exposure duration, we examined the mRNA expression levels of Nqo1-AS1 and Nqo1 in lung tissues of mice as well as in the mle-12 cells, which were exposed to CS or CSE for different durations. We observed that both Nqo1-AS1 and Nqo1 mRNA levels were remarkably upregulated in lung tissues of mice exposed to CS for 1week, 1 month and 3 months, and the fold changes of these two genes between mice exposed to CS and control animals were gradually increased along with the prolongation of CS exposure. Similarly, Nqo1-AS1 and Nqo1 mRNA expressions were also enhanced along with the increase of CSE concentration whereas decreased with the decrease of the CSE exposure duration. Furthermore, the Nqo1 protein level was also enhanced along with the increase of CSE concentration and the CSE exposure duration. Together with these findings, our data imply that Nqo1-AS1 (or its human homologue) and Nqo1 mRNA expression levels are increased with the increase of CS exposure, and both Nqo1-AS1 and Nqo1 mRNA expression levels are positively correlated with each other under CS exposure.

Nqo1 has been reported to be a multifunctional antioxidant enzyme, which plays critical roles in protecting cells from oxidative damage through proteasomal degradation, xenobiotic detoxification, regulation of p53, superoxide scavenging and the maintenance of endogenous antioxidants (Zhu et al., 2020). Furthermore, Nqo1 promotes Serpina1 mRNA translation, whose absence is involved in the pathogenesis of COPD (Di Francesco et al., 2016). Thus we speculated that Nqo1-AS1 might exert an effect on the CS-induced oxidative stress through regulating the expressions of Nqo1 and Serpina1. As expected, we found that Nqo1-AS1 overexpression enhanced the mRNA and protein levels of Nqo1 and Serpina1 mRNA expression in mle-12 cells, and attenuated CSE-induced oxidative stress (GSH, MDA and ROS). On the contrary, knockdown of Nqo1-AS1 significantly decreased Nqo1 at mRNA and protein levels as well as Serpina1 mRNA expression, and aggravated CSE-induced oxidative stress (GSH, MDA and ROS). Therefore, we concluded that Nqo1-AS1 is able to attenuate CS-induced oxidative stress through regulating the expression of Nqo1 in me-12 cells. Taken together, these findings clearly suggest that Nqo1-AS1 might exert its antioxidant effect by regulating Nqo1 expression.

Since NATs are capable of binding to their corresponding sense transcripts thereby regulating the expression of the latter (Jadaliha et al., 2018), we are interested in whether Nqo1-AS1 regulates the expression of Nqo1 through antisense pairing with Nqo1 mRNA. Interestingly, we observed that Nqo1-AS1 upregulated Nqo1 expression through binding to Nqo1 3′UTR and increasing Nqo1 mRNA stability.

The strengths of our study include determining the characterization of Nqo1-AS1, investigating the expression patterns of Nqo1-AS1 (or its human homologue) and Nqo1 in lung tissues of mice exposed to CS, mle-12 cells treated with CSE and PBMCs from patients with COPD, and examining the role of Nqo1-AS1 in the regulation of CS-induced oxidative stress. However, there are still some limitations in our study. An important limitation of our study is that we detected the expression levels of Nqo-AS1 and Nqo1 in PBMCs from patients with COPD and healthy donors, rather than those in lung tissues from patients with COPD and healthy donors. Undoubtedly, it would be better to detect the expressions of Nqo-AS1 and Nqo1 in lung tissues from patients with COPD and healthy donors, and analyze the correlations between smoking history and the expressions of Nqo-AS1 and Nqo1. However, as we all know, it is very difficult to obtain lung tissues from patients with COPD or healthy donors. In fact, PBMCs from patients with COPD and healthy donors are widely used in studies about CS-induced COPD. For example, Shen, W. et al. detected the mRNA expression levels of MBD2, miR-301a-5p, CXCL12 and CXCR4 in PBMCs from healthy controls and patients with stable COPD or with an acute exacerbation of COPD, and found that the MBD2/miR-301a-5p/CXCL12/CXCR4 pathway was shown to affect the migration of lung fibroblasts and monocyte-derived macrophages, which may play an important role during COPD exacerbations (Shen et al., 2020). Zhong, S. et al. analyzed hsa-miR-664a-3p and FHL1 mRNA expressions both in lung tissues from smokers with COPD and normal smokers from the GEO dataset GSE38974 and PBMCs from smokers with COPD and normal smokers, and found that the expression trends of hsa-miR-664a-3p and FHL1 in PBMCs from smokers with COPD were both consistent with those in lung tissues of smokers with COPD from the GEO dataset GSE38974, which demonstrating that the axis of hsa-miR-664a-3p and FHL1 might play a key role in CS-induced COPD (Zhong et al., 2019). Recently, the impacts of CS on both innate and adaptive immunity cells such as T lymphocytes, B lymphocytes, monocytes and macrophages have been widely discussed, which are the main components of PBMCs (Qiu et al., 2017). Thus, it is reasonable to speculate that the aberrant expression patterns of Nqo-AS1 and Nqo1 mRNA in PBMCs from patients with COPD might represent the CS-induced oxidative damage to innate and adaptive immunity cells of patients with COPD to some extent. Simultaneously, it is convincing that the smoking history of patients with COPD are correlated with the expression level of Nqo-AS1 or Nqo1 mRNA in their PBMCs.

In summary, our work demonstrated that Nqo1-AS1 (fantom3_F830212L20) oriented in antisense direction with respect to Nqo1, which is mainly located in the cytoplasm of mouse alveolar epithelium and had a very low protein coding potential. Nqo1-AS1 and Nqo1 mRNA expressions were increased with the increase of CS exposure. Nqo1-AS1 alleviated CS-induced oxidative stress by upregulating Nqo1 expression through antisense pairing withNqo1 3′UTR and increasing Nqo1 mRNA stability. Thus, our findings demonstrate that Nqo1-AS1 might play a critical role in inhibiting CS-induced oxidative stress, and may serve as a pivotal therapeutic target for COPD.

## Data Availability

The original contributions presented in the study are included in the article/supplementary material, further inquiries can be directed to the corresponding authors.
